# Anomalous wet summers and rising atmospheric CO_2_ concentrations increase the CO_2_ sink in a poorly drained forest on permafrost

**DOI:** 10.1073/pnas.2414539121

**Published:** 2024-10-25

**Authors:** Masahito Ueyama, Hiroki Iwata, Hirohiko Nagano, Naoki Kukuu, Yoshinobu Harazono

**Affiliations:** ^a^Department of Environmental Sciences and Technology, Graduate School of Agriculture, Osaka Metropolitan University, Sakai 599-8531, Japan; ^b^International Arctic Research Center, University of Alaska Fairbanks, Fairbanks, AK 99775-7340; ^c^Department of Environmental Science, Faculty of Science, Shinshu University, Matsumoto 390-8621, Japan; ^d^Institute of Science and Technology, Niigata University, Niigata 950-2181, Japan; ^e^Wood River Field Services, Fairbanks, AK 99709

**Keywords:** boreal forest, CO_2_ budget, permafrost, hydrological cycle, CO_2_ fertilization effect

## Abstract

At the northern high latitudes, rapid warming, associated changes in the hydrological cycle, and rising atmospheric CO_2_ concentrations, [CO_2_], are observed at present. Under rapid environmental changes, it is important to understand the current and future trajectories of the CO_2_ budget in high-latitude ecosystems. In this study, we present the importance of anomalous wet conditions and rising [CO_2_] on the long-term CO_2_ budget based on two decades (2003–2022) of quasicontinuous measurements of CO_2_ flux at a poorly drained black spruce forest on permafrost peat in interior Alaska. The long-term CO_2_ budget for the black spruce forest was a small sink of −53 ± 63 g C m^−2^ y^−1^. The CO_2_ sink increased from 49 g C m^−2^ y^−1^ for the first decade to 58 g C m^−2^ y^−1^ for the second decade. The increased CO_2_ sink was attributed to an 11.3% increase in gross primary productivity (GPP) among which 9% increase in GPP was explained by a recent increase in precipitation. Furthermore, a 3% increase in GPP in response to a 37-ppm increase in [CO_2_] was estimated from the data-model fusion. Our study shows that understanding the coupling between hydrological and carbon cycles and the CO_2_ fertilization effect is important for understanding the current and future carbon budgets of high-latitude ecosystems in permafrost regions.

## Significance

The CO_2_ budget of high-latitude ecosystems could change with rapid warming, associated changes in the hydrological cycle, and rising atmospheric CO_2_ concentrations. Based on 20 years of observations, we present a long-term increase in the CO_2_ sink during two decades in a black spruce forest in the permafrost region of interior Alaska. The current increase in a CO_2_ sink was associated with increased photosynthesis due to uncommon wet conditions and increased photosynthesis rates stimulated by rising atmospheric CO_2_ concentrations. We emphasize the importance of coupling hydrological and carbon cycles and the CO_2_ fertilization effect for understanding the current and future trajectories of high-latitude CO_2_ budgets.

Permafrost occupies approximately 11% of the global land surface (1) and is now susceptible to rapid environmental changes associated with high-latitude warming (2). High-latitude ecosystems are characterized by low-temperature conditions, a short vegetation growing season, and poor nutrient conditions owing to slow mineralization (3). Owing to the shallow active layer depth and low evapotranspiration, permafrost-dominated ecosystems often have poorly drained conditions with standing water (4). Such cold and/or wet conditions historically facilitated the accumulation of organic carbon in the peat, but rapid environmental changes might have turned carbon sinks into sources in recent years (5–7). A clear understanding of the changes in the carbon budget occurring in permafrost regions under the current environmental changes is of fundamental importance for predicting future climate changes.

Northern high latitudes are currently experiencing rapid warming and associated environmental changes. Surface air temperatures warmed 0.73 °C per decade from 1971 to 2019 over latitudes above 60°N (8), which was more than twice as fast as global warming. Warming leads to a wetter atmosphere because of moisture transport from low latitudes and increased convection (9, 10). Consequently, precipitation has recently intensified in high-latitude regions, where record-breaking precipitation has been measured at several areas, such as in interior Alaska (11) and Siberia (12). Under future climate scenarios, warming and wetting are predicted to intensify (9).

Wet conditions influence the ecosystem functions in permafrost regions. Consecutive wet years caused root rot in a Siberian larch forest in the permafrost zone, resulting in changes in vegetation structure and reduced carbon sink (13). In a poorly drained black spruce forest in a nonpermafrost peatland in boreal Canada, wetting conditions increased the CO_2_ sink associated with decreased heterotrophic respiration (14). In contrast, shifting wet conditions induced the thawing of permafrost in northern peatlands (15), resulting in sustained CO_2_ and CH_4_ emissions (2).

Continuous monitoring of the CO_2_ budget has been conducted in the Arctic and boreal zones (7, 16). Previous studies revealed that the annual CO_2_ sink decreased in response to autumn warming and prolonged autumn conditions (17), the CO_2_ sink increased in response to spring warming (18), the importance of CO_2_ emissions in winter (19), productivities increased due to the CO_2_ fertilization effect (20, 21), and spatial variability in CO_2_ fluxes (22). Although the number of monitoring sites has increased in the region and a monitoring network has been developed (16, 22), long-term observations over two decades have not yet reported for boreal forests on permafrost to our knowledge. Consequently, current monitoring networks cannot fully explain how the CO_2_ budget of high-latitude ecosystems responds to ongoing rapid warming on a decadal timescale (23).

Here, we report the importance of hydrological conditions for explaining decadal-scale variations in the CO_2_ budget in a poorly drained black spruce forest in permafrost peat based on two-decade-long quasicontinuous observations from 2003 to 2022 in interior Alaska. The observations consisted of turbulent fluxes by eddy covariance measurements, soil respiration, and meteorological and environmental variables. Furthermore, an increase in the CO_2_ sink associated with a rising atmospheric CO_2_ concentration, [CO_2_], was estimated using a data–model fusion approach (20, 21). Black spruce forests occupy a large area of the landscape in boreal North America and thus play an important role in carbon, water, and energy exchange in boreal North America. The results obtained in this report could suggest the future trajectory of permafrost carbon feedback.

## Results and Discussion

### Anomalous Warm and Wet Climate in Recent Years.

**Fig. 1.**
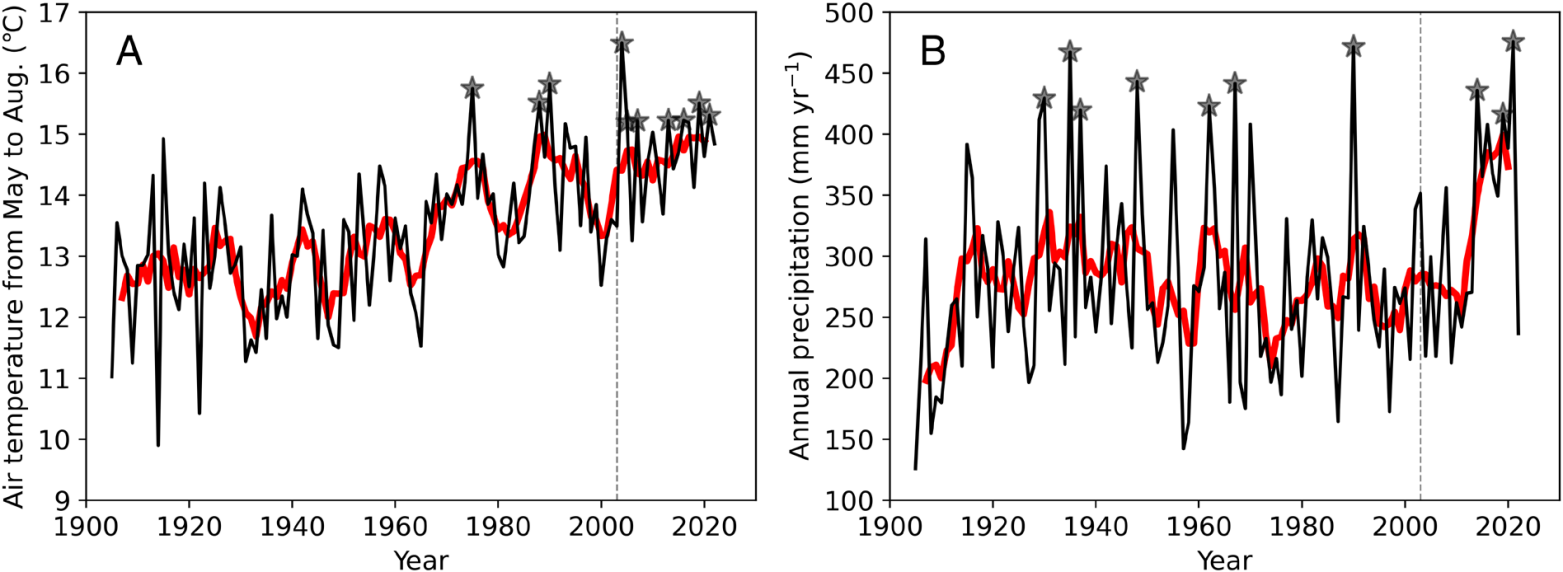
Air temperature during the growing season from May to August (*A*) and annual total precipitation (*B*) at Fairbanks International Airport according to the National Weather Service. The black lines represent values for individual years, the red lines represent 5-y moving means, and the stars represent values in the top 10 y from 1900 to 2022. The vertical dashed line represents the year 2003 when the measurement started.

The five-year moving mean of air temperature and precipitation showed that the entire study period was a warm period, with the wettest period occurring in the second half of the study period (Fig. 1). The mean air temperature from May to August was 12.7 °C from 1905 to 1924, 13.2 °C from 1951 to 1970, and 14.7 °C from 2003 to 2022. Seven of the top ten temperature records were measured during the 2003–2022 study period. The annual precipitation increased from 1905 to 2022, when three of the top ten precipitation years (2014, 2019, 2021) were recorded during the study period, and 2021 had the highest annual precipitation in the record. By separating the precipitation for the growing season (May to September) and cold period (October to April), it was found that wet conditions were associated with both increased rainfall and snowfall (*SI Appendix*, Fig. S1).

**Fig. 2.**
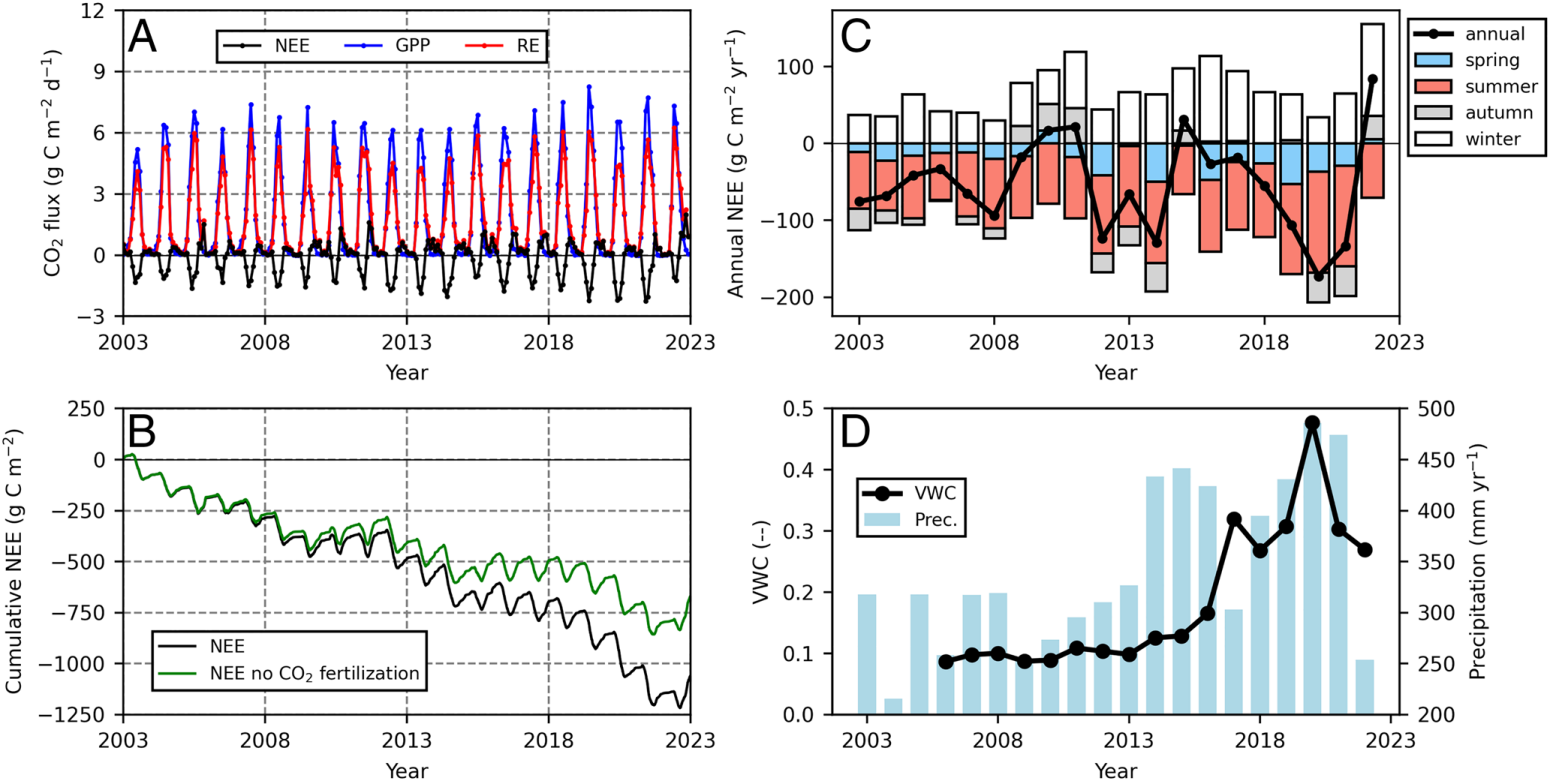
Monthly mean NEE, GPP, RE (*A*), cumulative NEE (*B*), annual NEE (*C*), and volumetric water content (VWC) at 0 to 10 cm for June and July and annual precipitation (*D*) from 2003 to 2022. The CO_2_ fertilization effect was estimated based on the biochemical photosynthesis model whose parameters were constrained by GPP by the eddy covariance measurements (*B*). The NEE was calculated as the difference between the RE and GPP, where the estimated CO_2_ fertilization effect was eliminated from the GPP. Spring was from April to May, summer was from June to July, and autumn was from August to September, and winter was from October to March.

In association with the high precipitation, the volumetric water content (VWC) at 0 to 10 cm was unusually high starting in 2014 or 2017, depending on the location (Fig. 2*D* and *SI Appendix*, Fig. S2). The increased VWC at one of two locations was delayed by two years after the high precipitation years, possibly owing to the effect of microtopography. Lags in hydrological conditions are often reported in permafrost regions (13, 24) because rainwater in the late growing season is stored in frozen soil during winter and ice in the bottom of the active layer is stored until the late growing season of the next year (25).

Besides wetting, other environmental variables, such as the annual air temperature (*SI Appendix*, Fig. S3*A*), start of the growing season (*SI Appendix*, Fig. S3*C*), end of the growing season (*SI Appendix*, Fig. S3*D*), growing season length (*SI Appendix*, Fig. S3*E*), and thaw depth (*SI Appendix*, Fig. S3*H*), showed no trends during the decades. The soil temperature at 10 cm increased by 1.6 °C, and an 8.5-cm deepening in thaw depth was observed during the wet years (2016–2021), possibly due to the increased thermal conductivity of the soil under wet conditions.

### Long-Term Carbon Fluxes.

The long-term CO_2_ budget for the black spruce forest was a carbon sink of −53 ± 63 g C m^−2^ y^−1^ from 2003 to 2022 (pulse/minus denotes SD for each year) with the large interannual variations (Fig. 2 *A*, *C*). The measured sink was nearly double compared to the mean global peat accumulation on a given growing degree day (1780 degree days) (26). The CO_2_ sink increased from 49 g C m^−2^ y^−1^ for the first decade to 58 g C m^−2^ y^−1^ for the second decade (Fig. 2*B*), although there was no trend in the annual NEE owing to the large interannual variations. Until 2011, the CO_2_ budget gradually shifted from a CO_2_ sink to a source, which was caused by increased ecosystem respiration (RE) (Fig. 2*C*) associated with an anomalous increase in the autumn temperature (17). Anomalous autumn warming was detected from 2009 to 2011. Thereafter, the forest turned into a CO_2_ sink, and the magnitude of the sink tended to increase until 2021. The increased sink was mostly attributed to greater photosynthesis in the summer months. Monthly mean NEE showed a significant negative trend in June and July (*SI Appendix*, Fig. S5 *F* and *G*). During the second decade, the magnitudes of the CO_2_ sinks in summer and spring were greater than those in the first decade (Fig. 2*C*). The alternative CO_2_ budget by a different gap-filled method showed a qualitatively-consistent sink of −33 ± 51 g C m^−2^ y^−1^ (*SI Appendix*, *A2* and *SI Appendix*, Fig. S4).

**Fig. 3.**
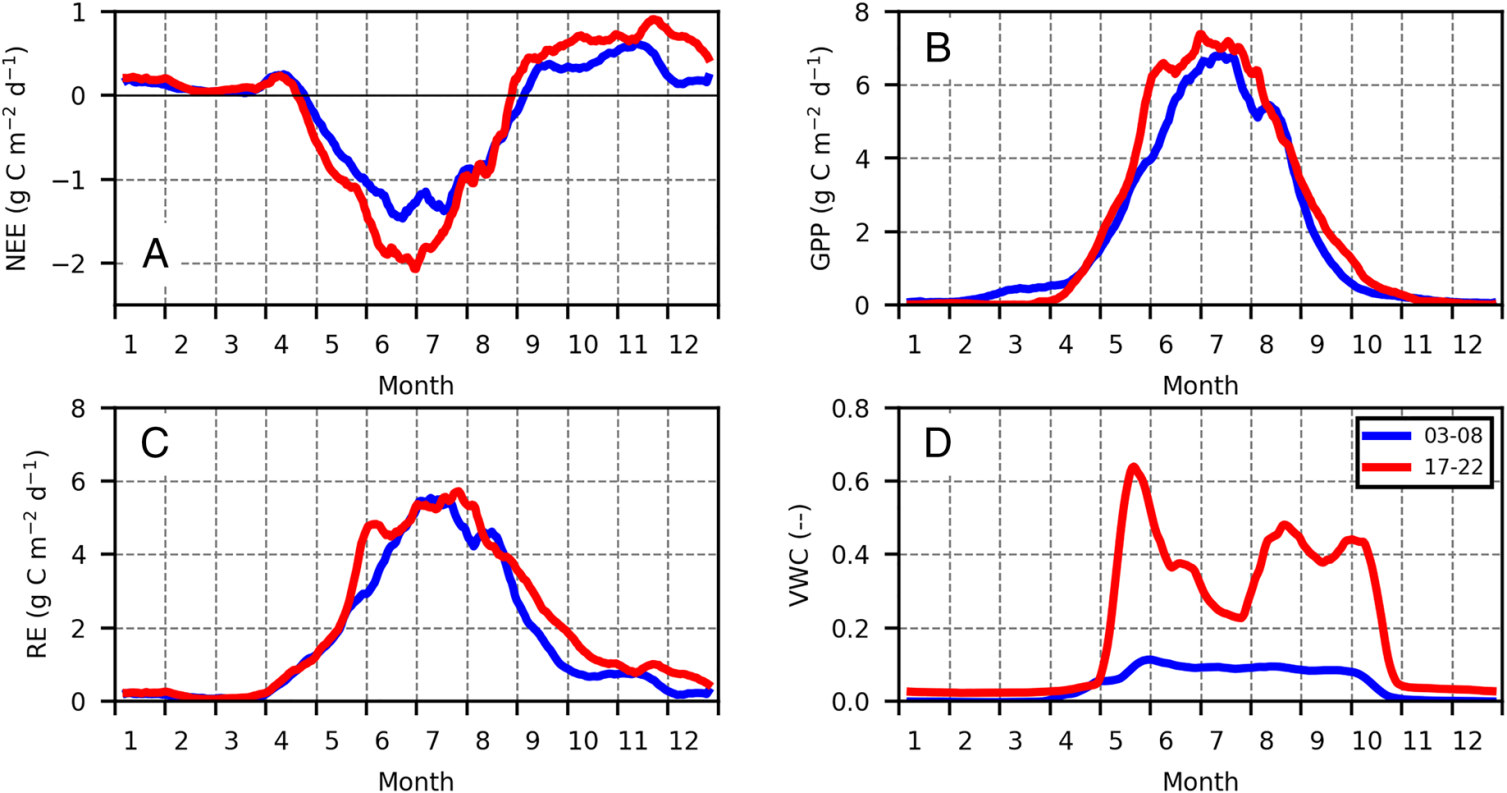
Mean seasonality in NEE (*A*), GPP (*B*), RE (*C*), and volumetric water content at the 0 to 10 cm depth (*D*). The gray lines represent data from 2003 to 2008 and the black lines represent data from 2017 to 2022.

The mean seasonality in the CO_2_ flux showed that the summer CO_2_ sink was nearly doubled in recent years compared to that in the mid-2000s (Fig. 3*A*). The greater sink was explained by changes in GPP (Fig. 3*B*). The annual GPP significantly increased (9.2 g C m^−2^ y^−2^; *P* = 0.01), where GPP increased 11.3% from the first to the second decade. The annual RE increased at a smaller rate,10.7%, during the two decades, but did not show a significant positive trend possibly due to large interannual variation. Seasonal maximum of light-saturated photosynthesis significantly increased during the two decades (0.20 µmol m^−2^ s^−1^ y^−1^; *P* < 0.01) (*SI Appendix*, Fig. S3*I*), but a positive trend in nighttime RE was not detected (*SI Appendix*, Fig. S3*K*). This increase in the summer photosynthesis could be explained by a significant increase in the seasonal maximum leaf area index (0.03 y^−1^; *P* = 0.01) (*SI Appendix*, Fig. S3*F*). Based on photos taken from the tower, black spruce trees were greatly growing for the areas of the dominant wind direction during the two decades (*SI Appendix*, Fig. S6), which could contribute to the increased summer photosynthesis. The increased and grown trees could explain the increased photosynthesis and CO_2_ sink.

**Fig. 4.**
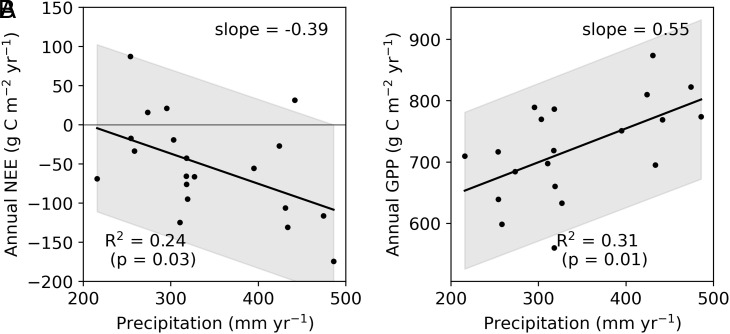
Relationships between annual precipitation and NEE (*A*) and between annual precipitation and annual GPP (*B*).

Interannual variations in NEE were explained by variations in the annual total precipitation (Fig. 4*A*). Greater precipitation induced a greater CO_2_ sink in the forest (−0.39 g C m^−2^ y^−1^ mm^−1^; R^2^ = 0.24; *P* = 0.03). Correlation to the annual precipitation was significant for the annual GPP (Fig. 4*B*) but not for the annual RE. The decreased CO_2_ sink or GPP in dry years was also reported in a black spruce forest in interior Alaska (27), possibly owing to drought stress of vegetation (28, 29). Wetting could alleviate drought conditions. The response to water conditions is a common feature of black spruce forest or trees, where increased productivities by wetting and decreased productivities by drying were reported based on the long-term eddy covariance measurements and tree ring analyses (*SI Appendix*, Table S1). Increased cambial activity by alleviating water deficit was also evident in the black spruce at this site (30) but is also a known response of a wide range of trees (31). This response suggests that increased precipitation increased a carbon sink capacity by vegetation, resulting in an increased source capacity (i.e., increased photosynthesis and increased LAI; *SI Appendix*, Fig. S3 *F* and *I*) for alleviating source–sink decoupling (31). Based on the relationship between GPP and precipitation (Fig. 4*B*), GPP increased by 8.2% in response to the increased precipitation from the first to the second decade, which was 3.1% smaller magnitude than the actual increase, 11.3%, during the two decades. Insignificant correlation between RE and precipitation could be explained by reduced heterotrophic respiration under anoxic conditions. The recent increase in precipitation could increase anoxic fraction in the active layer. Soil respiration greatly decreased with increasing VWC at the 0 to 10 cm depth in a plot covered by *Sphagnum* moss (*SI Appendix*, Fig. S7*A*), which was the major understory within the flux footprint. Similar responses were also observed for soil respiration in *Carex* plots (*SI Appendix*, Fig. S7 *B* and *C*), but the responses were weak. Reduced RE by wetting was previously reported in black spruce forests on nonpermafrost soil (14, 18), indicating that a no increase in RE and an increase in CO_2_ uptake resulting from increased soil water conditions are common characteristics of black spruce forests irrespective of the occurrence of permafrost. Thus, the decrease in soil respiration could compensate for increased aboveground respiration associated with increased GPP, resulting in insignificant change in RE in terms of increased precipitation.

The study site became a CO_2_ source in 2022, which was caused by 20-d delayed spring onset due to delayed snowmelt compared with the 20-y mean and lowest summer precipitation (89 mm from May to August) during the study period. For the cold season, the NEE in December tended to show a weak positive trend (*P* = 0.02; and *SI Appendix*, Fig. S5), possibly because of prolonged zero-curtain durations at the 25 cm soil depth in this forest (32). During the second decade, VWCs in November and December were higher than those in the first decade, suggesting that the soil took longer times to freeze completely.

Interannual variations in annual NEE were associated with variations in RE rather than GPP (*SI Appendix*, Fig. S8 *A* and *B*), indicating that high interannual variabilities in RE determined year-to-year variations in the CO_2_ budget. Interannual variations in RE explained more of the NEE than did those in GPP for the autumn, and winter, but those in GPP explained more for the spring and summer (*SI Appendix*, Fig. S8). This result was in contrast to the cause of the decadal change in NEE. Namely, GPP could shift stronger than RE at the decadal timescale, but at the shorter timescale, RE varied more than GPP. The weaker increase in RE might be caused by insignificant warming during the decades (Fig. 1*A* and *SI Appendix*, Fig. S3), resulting in relatively stable decomposition and plant respiration over time.

### Effect of Rising Atmospheric CO_2_ Concentrations.

Based on the data model fusion (*Materials and Methods* and *SI Appendix*, *A3*), rising [CO_2_] strongly stimulated GPP (*SI Appendix*, Fig. S9), increasing the CO_2_ sink from −671 g C m^−2^ to −1,060 g C m^−2^ during the two decades compared with that in which the CO_2_ effect was not considered (Fig. 2*B*). This stimulation is known as the CO_2_ fertilization effect and was estimated via a biochemical photosynthesis and stomatal conductance model (33, 34) coupled with sun/shade radiation transfer (35). The necessary parameters for estimating CO_2_ fertilization, namely, the canopy-integrated maximum carboxylation rate and stomatal behavior, were estimated at the daily timescale using GPP, turbulent fluxes, [CO_2_], and environmental variables observed at the site (20, 21). The data-model fusion approach could allow us to evaluate the CO_2_ fertilization effect, by directly inputting observed data into well-established ecophysiological models (*SI Appendix*, *A3*) by minimizing uncertainties associated with complex ecosystem processes. [CO_2_] in the summer increased from 378 ppm in 2003 to 415 ppm in 2022 (*SI Appendix*, Fig. S9*B*). These increases in [CO_2_] could theoretically increase GPP in 2022 by 5.7% compared to that in 2003 (*SI Appendix*, Fig. S9*A*). Subtracting the stimulated CO_2_ fertilization effect from GPP decreased the CO_2_ sink for two decades, especially in the second decade of the study period (Fig. 2*B*); it is worth noting that the increased CO_2_ sink here is the potential maximum, since we assumed that RE did not increase due to the CO_2_ fertilization. Mean stimulation by the CO_2_ fertilization was 7.5 g C m^−2^ y^−1^ in the first decade and 27.8 g C m^−2^ y^−1^ in the second decade, which contributed to 3.0% increase in GPP during the two decades. This increase was of similar magnitude to the difference in the decadal increases in GPP associated with wetting (8.2%) and the actual increase (11.3%).

Estimated canopy-integrated or leaf-scale carboxylation rates at 25 °C did not decrease during the two decades (*SI Appendix*, Fig. S3 *M* and *N*). These results indicated that the downregulation of photosynthesis could not be detected under the given GPP and environmental conditions. Previous experimental studies using CO_2_ enrichment indicated the productivity of black spruce trees increased with enhanced [CO_2_], but this was not clear for understory shrubs, forbs, and graminoids (summarized in *SI Appendix*, Table S2). For tree species, light compensation points were decreased under enhanced [CO_2_] (36), which could contribute to increased productivity. A mesocosm study showed that enhanced [CO_2_] increased the productivity of *Sphagnum* mosses. Consequently, black spruce trees and mosses could potentially respond to rising [CO_2_] in this forest, although long-term CO_2_ enrichment studies are required for identifying important species for the CO_2_ fertilization effect.

Intrinsic water use efficiency (iWUE) increased over time, even if the effects of rising [CO_2_] were not taken into account. Although stomatal responses are known to be optimally regulated to respond to photosynthetic rate, atmospheric humidity, and CO_2_ concentration (34), the index of stomatal response to environmental conditions (m_bb_; *SI Appendix*, Fig. S3*L*) has decreased significantly over the past 20 years (p < 0.01). This decrease indicates a shift toward a more stomatal-closed ecosystem in the same environment. The decreased m_bb_ might be explained by the increased black spruce trees over time (*SI Appendix*, Fig. S6), because m_bb_ of black spruce was lower than that of understory and mosses (37). In addition to decreased m_bb_, rising [CO_2_] enhanced iWUE (21, 38). The increased iWUE through these two processes could alleviate water stress for black spruce that is a water-limiting vegetation (*SI Appendix*, Table S1) and thus contributed to increased GPP and CO_2_ sink.

## Conclusion

Two decades of measurements clearly showed the CO_2_ sink increased with increased productivity. The most of this increase was attributed to the wetting and associated vegetation growth in recent years, showing that close coupling between hydrological and carbon cycles in a poorly drained forest on permafrost peat. Anomalous wet conditions did not stimulate ecosystem respiration, possibly due to reduced peat decomposition under anoxic conditions (4), but increased GPP. Treed bogs on permafrost are known to emit CH_4_ at very low levels because of low water table conditions and low temperatures at the bottom of the active layer (39). Like other treed bogs on permafrost, this forest acted as a very small CH_4_ source even in wet years (40). Consequently, wetter conditions increase the carbon sink capacity in poorly drained forests on permafrost.

High-latitude warming is expected to intensify in the future (8) and increase precipitation (9). Recently, record-breaking high precipitation events have been reported in many high-latitude lands (12), including interior Alaska (11). The two decades of measurements reported in this study suggest that such high precipitation has negative feedback effects on further warming through increases in carbon and greenhouse gas sinks. However, continued wet and warm conditions might collapse permafrost owing to increased thermal conduction (41) and this will be associated with changes in vegetation (27). A deepening in the active layer was observed in wet years in this forest. The integrity of permafrost and its consequences for the carbon budget should be monitored via long-term observations. The decadal increases in GPP during summer and RE in winter obtained in this study are consistent with the state-of-the-art eddy covariance synthesis using 70 permafrost and nonpermafrost ecosystems (7). In contrast, the current measurement indicated an increased CO_2_ sink during the two decades, although the synthesis study showed a decadal decrease in CO_2_ sink for permafrost ecosystems. As there are no reports of two-decade-long measurements in a boreal forest on permafrost, further long-term monitoring of CO_2_ fluxes across multiple ecosystems will help ensure whether the current responses and trends are representative of permafrost forests.

The effect of rising [CO_2_] was estimated to be the most important environmental factor affecting the long-term CO_2_ budget, based on the data–model fusion approach (21). Current results indicate that obtaining accurate estimates of the CO_2_ fertilization effect is a high priority and a challenge for understanding future carbon budgets at high latitudes because the effect has not been well characterized via observation. Experiments, such as the Free-Air CO_2_ Enrichment (42), in permafrost forests could help constrain the CO_2_ fertilization effect.

## Materials and Methods

### Site Descriptions.

The study site was located in an open black spruce forest on ice-rich permafrost in interior Alaska (64°52’N, 147°51’W, elevation 155 m; US-Uaf registered in AmeriFlux). The dominant overstory was black spruce (*Picea mariana*) with a tree density of 4,500 tree ha^−1^ and a mean tree height of approximately 3 m. Based on tree-ring analysis, the tree age ranged from 36 to 119 years with the median of 91 years in 2012. The forest floor was completely covered by understory species and sphagnum mosses, including *Betula glandulosa*, *Ledum groenlandicum*, *Vaccinium ulginosum*, *Vaccinium vitis-idea*, *Rubus chamaemorus*, *Larix laricina, Carex* spp., and *Alnus crispa*. The mean annual maximum leaf area index (LAI) by a plant canopy analyzer (LAI-2000; LI-COR, USA) was approximately 2 m^2^ m^−2^. The carbon content was approximately 44 kg C m^−2^ in the top 1-m soil, among which 23 kg C m^−2^ was stored in peat layer (43). Detailed information on the site is available in published studies (17, 25, 40). The mean annual air temperature at 2 m was −3.8 °C and the annual total precipitation was 342 mm y^−1^ during the study period from 2003 to 2022. Approximately one-third of the precipitation fell as snow; snow precipitation was based on measurements at the Fairbanks International Airport (approximately 5.5 km south of the study site) by the National Weather Service.

### Measurements.

Fluxes of CO_2_, water vapor, and sensible heat have been measured with the eddy covariance method since October 2002 (17). A sonic anemometer (CSAT3, Campbell Scientific Inc.) and open-path gas analyzer (LI-7500, LI-COR) were installed at 6 m above the ground. Turbulent fluctuations were measured at 10 Hz using a datalogger (CR3000, Campbell Scientific Inc.). We also measured ancillary environmental variables, including air temperature, relative humidity, upward and downward shortwave and longwave radiation, photosynthetically photon flux density, soil temperature and volumetric water content (VWC) at multiple depths, and water table depth, using a datalogger (either CR3000 or CR1000, Campbell Scientific Inc.). The vertical CO_2_ concentration profile at 1, 2, 4, and 8 m were measured to determine the storage flux. Soil respiration and CH_4_ fluxes were measured with the automated closed chamber technique with a laser-based analyzer (Greenhouse Gas Analyzer, Los Gatos Research Inc., USA) during the snow-free period from 2016 to 2018 at four locations on the forest floor: one for *Sphagnum* moss, two for *Carex* spp., and one for lichen (40). Details for the ancillary measurements are available in *SI Appendix*, *A1*.

Thaw depth and LAI were manually measured approximately twice a month during the growing season. Thaw depth was measured by inserting a bless rod at ten locations, whereas LAI was measured with a plant canopy analyzer (LAI-2000, LI-COR) at eight locations.

## Data Processing

Turbulent fluxes were calculated with the eddy covariance method with necessary corrections (shown in *SI Appendix*, *A2*). Then, net ecosystem exchange (NEE) was calculated as the sum of the measured CO_2_ fluxes and the storage flux. We removed the data obtained from the low turbulent conditions with a threshold of the friction velocity (0.1 m s^−1^) or instationary conditions. We also removed the data when 80% flux footprint exceeded the fetch for the black spruce forest. The partitioning of CO_2_ flux into gross primary productivity (GPP) and ecosystem respiration (RE) was conducted based on the nighttime-based approach (44, 45). We evaluated the uncertainties associated with gap-filling procedures by comparing NEE filled with two different methods: commonly used combined look-up-table (LUT) and nonlinear regression (NLR) and random forest regression. Since there were no clear differences between the two gap-filled NEE, we used NEE filled by the combined LUT and NLR methods in the main text. Details for the processing of the eddy covariance method, the gap-filling flux, and partitioning are available in *SI Appendix*, *A2*.

We eliminated negative daily mean CO_2_ fluxes during the cold period and filled the gaps with estimated RE (17) because artificial negative fluxes were observed with an open-path gas analyzer (46, 47). Since the empirical correction for the artifact (46, 47) could induce systematic bias at this site (44), we eliminated the negative daily mean fluxes during the cold season when the soil temperature at 2 cm was less than 1 °C (17), assuming that photosynthesis is regulated via soil water availability by soil thaw (18, 48). The artificial flux accounted for 34 g C m^−2^ y^−1^ over 20 y.

The decadal trend was estimated based on the Theil-Sen slope, and the significance was evaluated based on the Mann–Kendall test with a significance level of 0.05. The trend analysis was performed with the pymannkendall library (version 1.4.2) in Python.

### Evaluating CO_2_ Fertilization Effects.

The effects of CO_2_ fertilization were evaluated via the sun/shade model (iBLM-EC version 2.0; 21), which couples processes for biochemical photosynthesis (33), stomatal conductance (34), radiative transfer (35), and leaf boundary layer. The iBLM-EC inversely estimated the important ecophysiological parameters, namely, canopy-integrated maximum carboxylation and electron transport rate and slope between photosynthesis and stomatal conductance, at the daily timescale through the input of the following measured variables: GPP, sensible heat flux, latent heat flux, friction velocity, LAI, [CO_2_], and relevant micrometeorology. We used the scheme (49) for the Farquhar photosynthesis model. Once the actual parameters for the CO_2_ fertilization process (CO_2_ demand and supply functions of photosynthesis; 50) were constrained for each day, changes in canopy photosynthesis, namely, GPP, were calculated by inputting different [CO_2_] data into the constrained model. First, as a control experiment, the GPP was calculated with the transient [CO_2_] from 2003 to 2022. For consistent [CO_2_] values during two decades, we corrected [CO_2_] with data measured by an aircraft at Poker Flat near Fairbanks, operated by the National Oceanic and Atmospheric Administration (51). We corrected the [CO_2_] to be consistent between daytime mean CO_2_ concentrations at the site and those at aircraft altitudes less than 2000 m at the monthly timescale. Then, the GPP was calculated with constant [CO_2_] as an input, in which the measured [CO_2_] in each year was adjusted at the monthly timescale to match the aircraft measurements in 2003 instead of those in each year. The difference in GPP between transient and constant [CO_2_] represented the change in GPP by rising [CO_2_], namely, the CO_2_ fertilization effect. The estimated parameters, downscaled to the leaf level, were consistent with those obtained in leaf-chamber measurements (37). Since the ecophysiological parameters were only estimated using non-gap-filled fluxes, uncertainties in gap-filling were not propagated to parameter estimating and the inferred CO_2_ fertilization effect. Further details on the methods used to estimate CO_2_ fertilization and model structures have been descried previously (20, 21) and *SI Appendix*, *A3*.

## Supplementary Material

Appendix 01 (PDF)

## Data Availability

Observation data have been deposited in AmeriFlux (https://doi.org/10.17190/AMF/1480322) (52). The eddy covariance data at US-Uaf are available from the AmeriFlux database (52). All the measurement data are available in following database under CC By 4.0. The soil respiration by the chamber measurements are publicly available from the Arctic Data System (ADS; https://ads.nipr.ac.jp/data/meta/A20210930-001) (53). The code of the iBLM-EC model is available from the web site (https://www.omu.ac.jp/agri/ecolmet/ueyama/software/) (54).
