# Supplementary material for: Anomalous wet summers and rising atmospheric CO2 concentrations increase the CO2 sink in a poorly drained forest on permafrost
Source: Proc Natl Acad Sci U S A. 2024 Oct 25;121(44):e2414539121. doi: 10.1073/pnas.2414539121 (PMC11536150; doi:10.1073/pnas.2414539121)
Supplement: Supplementary file 1 — Appendix 01 (PDF) [file pnas.2414539121.sapp.pdf]

Supporting Information for

Anomalous wet summers and rising atmospheric CO_2 concentrations increase the CO_2 sink in a poorly-drained forest on permafrost

Masahito Ueyama[a, b], Hiroki Iwata[c,b], Hirohiko Nagano[d], Naoki Kukuu[e], Yoshinobu Harazono[b]

[a] Department of Environmental Sciences and Technology, Graduate School of Agriculture, Osaka Metropolitan University, Sakai 599-8531, Japan; [b] International Arctic Research Center, University of Alaska Fairbanks, Fairbanks, AK 99775-7340, USA; [c] Department of Environmental Science, Faculty of Science, Shinshu University, Matsumoto 390-8621, Japan; [d] Institute of Science and Technology, Niigata University, Niigata 950-2181, Japan; [e] Wood River Field Services, Fairbanks, AK 99709, USA

Corresponding Author: Masahito Ueyama

Email: mueyama@omu.ac.jp

This PDF file includes:

Figures S1 to S9
Tables S1 and S2
SI References

A1. Ancillary measurements

We also measured ancillary environmental variables, including air temperature, relative humidity, upward and downward shortwave and longwave radiation, photosynthetically photon flux density (PPFD), soil temperature and volumetric water content (VWC) at multiple depths, and water table depth, using a datalogger (either CR3000 or CR1000, Campbell Scientific Inc., USA). Air temperature and relative humidity were measured at 8, 4, 2, and 1 m using a temperature and humidity sensor (HMP45C or HMP46, Vaisala, Finland) with ventilated mounts. Four components of radiation were measured using (CNR4, Kipp & Zonen, Netherlands) since 2008. Until 2008, upward and downward shortwave radiation were measured using pyranometers (CMP3, Kipp & Zonen, Netherlands). Downward, upward and transmitted PPFD was measured using quantum sensors (LI-190, Li-Cor, USA). Soil temperatures were measured at depths of 0.1, 0.2, 0.5 and 0.8 m using thermocouple thermometers, and also measured at 0.02, 0.05, 0.1, 0.2, 0.3, 0.4, 0.5, 0.6 and 1 m using a thermistor (T107, Campbell Scientific Inc.). Using a time domain reflectometry (CS616, Campbell Scientific Inc.), VWC were measured at the two locations at the depth 0-10 cm and at the single locations at the depth of 0-30 cm. Water table depth was measured using pressure transducers (CS445 and CS450, Campbell Scientific Inc.) at three locations. Barometric pressure was measured using a pressure sensor (CS105, Vaisala). Rainfall was measured using a rain gauge (TE525, Campbell Scientific Inc.). Wind speed was measured at 8, 4, 2, and 1 m using a three-cup anemometer (VF016, Makino, Japan). Wind direction was measured at 10 m using a wind vane (03301, R. M. Young, USA). Ground heat flux was measured at three locations using (HFT3, Radiation Energy Balance Systems, USA).

Thaw depth and LAI were manually measured approximately twice a month during the growing season. Thaw depth was measured by inserting a bless rod at ten locations, whereas LAI was measured with a plant canopy analyzer (LAI-2000, LI-COR) at eight locations.

A2. Data processing of the eddy covariance method

Turbulent fluxes were calculated with the eddy covariance method using the Flux Calculator program (1). After the removal of raw data spikes, the covariance between the vertical wind velocity and scalar quantity was determined. We applied necessary corrections for the eddy covariance method (2), including coordinate rotation with the double rotation method, density correction (3), corrections for high-frequency loss (4), artificial fluctuations in the sonic air temperature due to water vapor (5), and the theoretical lag between the sonic anemometer and the open path gas analyzer (i.e., constant lag time due to signal processing difference between the sonic anemometer and the gas analyzer).

The partitioning of CO_2 flux into gross primary productivity (GPP) and ecosystem respiration (RE) was conducted with the Flux Analysis Tool program (1). First, net ecosystem exchange (NEE) was calculated as the sum of the measured CO_2 fluxes and the storage flux. We removed the data obtained from the low turbulent conditions with a threshold of the friction velocity (0.1 m s^{-1}) or instationary conditions. Although the threshold approach has been reported to not fully filter weak turbulent conditions, especially decoupling between above and below the canopy in winter (6), we believe that potential biases might be small because of the low mean canopy height (3 m) and the open canopy structure. We also removed the data when 80% flux footprint exceeded the fetch for the black spruce forest. Then, daily parameters for RE were determined with 28-day moving windows, where parameters for the so-called Q_{10} model were determined with 100 bootstrapping samples. Gap-filling of CO_2 fluxes was conducted with 1) a combined look-up-table (LUT) and non-linear regression (NLR) (7) and 2) a random forest regression. The data

availability for the CO_2 flux was 26% ± 5%, where the plus/minus sign denotes the standard deviation over the 20 years. Data availability was higher in the growing season (38%) than in the non-growing season (16%). Except for a long data gap in July and August 2004, there was no long data gap in the growing season, although snow on the sonic anemometer hampered measurements in some winters (Fig. S4b). A negative NEE represents a CO_2 sink, and a positive NEE represents a source.

The combined LUT and NLR was applied using the Flux Analysis Tool program (1). For the combined method, first, small gaps shorter than two hours were filled with linear interpolation. LUT was created using photosynthetically active radiation (PPFD), air temperature, and vapor pressure deficit (VPD) within a 15-day moving window. If gaps could not be filled with LUT, NLRs were used for filling NEE, where daytime NEE was filled with non-rectangular hyperbola function with 15-day moving window and nighttime NEE was filled with so-called Q_{10} function with 29-day moving window. The combined LUT and NLR methods were applied 100 times with bootstrapped samples, and then means of bootstrapping was used to fill the gap. Standard error of the bootstrapping was calculated as the random error of this method (2). The score for this method was 1.15 ± 0.24 μmol m^{-2} s^{-1} in root mean square error (RMSE) and 0.82 ± 0.04 in the determination coefficient (R^2), where the plus/minus sign represents the standard deviation of the score for each year.

As an independent gap-filled NEE, we prepared a NEE filled with the random forest regression. The input feature of random forest regression was PPFD, air temperature, and VPD, and the model was trained with a 3-year moving window. We applied 5 × 2 cross-validation with a random search to tune the hyper-parameter using the train data (8). The train data were 90% of the available data, and the other 10% were used as the test data. We repeated these processes 20 times to obtain regressions that had different hyperparameters from the 20 different initial parameters and then calculated the mean and standard error of the NEE. R^2 for the test data was 0.70 and that for training data was 0.78. We used a scikit-learn library (version 1.2.2) in Python 3.

Based on the comparison, the combined method provided greater long-term CO_2 sink (-53 g C m^{-2} yr^{-1} in NEE) than random forest regression (-33 g C m^{-2} yr^{-1} in NEE) (Fig. S4). The difference was 19 ± 35 g C m^{-2} yr^{-1}, where the plus/minus sign represents the standard deviation of the difference in each year. Although the mean NEE was different in the two methods, the interannual variability was similar (R^2 = 0.82). Consequently, we used the NEE filled by the combined method for consistency to our previous study (2).

We eliminated negative daily mean CO_2 fluxes during the cold period and filled the gaps with estimated RE (2) because artificial negative fluxes were observed with an open-path gas analyzer (9, 10). Since the empirical correction for the artifact (9; 10) could induce systematic bias at this site (1), we eliminated the negative daily mean fluxes during the cold season when the soil temperature at 2 cm was less than 1 °C (2), assuming that photosynthesis is regulated via soil water availability by soil thaw (11; 12). The artificial flux accounted for 34 g C m^{-2} yr^{-1} over 20 years.

A3. Model

For evaluating CO_2 fertilization effect, we used the data-model fusion approach that constrained the sun/shade canopy photosynthesis model (iBLM-EC version 2.0) (8) using observed data. The model couples processes for biochemical photosynthesis (13), stomatal conductance (14), radiative transfer (15), and leaf boundary layer. The iBLM-EC model integrated necessary processes for estimating CO_2 fertilization effect. Their important parameters, namely, canopy-integrated maximum carboxylation and electron transport rate and slope between photosynthesis and stomatal conductance, can be inferred at the daily timescale. The simple

structure and data-driven approach allowed ecophysiological responses inferred from data without solving ecological processes (e.g., allocation, decomposition, phenology, and nitrogen cycle) whose uncertainties often propagate to various ecological processes. Previously, the iBLM-EC model was validated in the study site, where estimated leaf-scale maximum carboxylation rate was consistent with those measured at the site (16). Although the full description of the iBLM-EC model is available in previous studies (8, 17), we briefly describe the model structure.

A3-1. Photosynthesis model

Based on the biochemical photosynthesis model (13), photosynthesis rate (A) is determined as the minimum rate of Rubisco-limited photosynthesis (A_v) and RuBP-limited photosynthesis (A_j):

$$A = min\left(A_v, A_j\right) - R_l \tag{S1}$$

$$A_v = Vc_{max} \frac{p_i - \Gamma_*}{p_i - K_c(1 + p_o/K_o)} \tag{S2}$$

$$A_j = J \frac{p_i - \Gamma_*}{4p_i + 8\Gamma} \tag{S3}$$

where R_l is mitochondrial respiration in light, Vc_{max} is the maximum carboxylation rate, J is the electron transport rate, Γ_* is the CO_2 compensation point for photosynthesis in the absence of mitochondrial respiration, p_i is intercellular CO_2 partial pressure, p_o is intercellular O_2 partial pressure, and K_c and K_o are Michaelis-Menten constants. In this study, we used the parameterization of the photosynthesis model (18). The variables Vc_{max}, J, K_c, K_o, Γ_*, and R_l change with leaf temperature, and are often parameterized with various types of kinetic functions.

The electron transport rate is described as follows:

$$J = \frac{I_{le} + J_{max} - \sqrt{(I_{le} + J_{max}) - 4 \times 0.9 \times I_{le} \times J_{max}}}{2 \times 0.9} \tag{S4}$$

$$I_{le} = 0.3 \times I_l \tag{S5}$$

where J_{max} is the maximum electron transport rate, I_{le} is PPFD effectively absorbed by PSII, and I_l is incoming PPFD.

Temperature dependence of Vc_{max} and J_{max} are calculated based on the Arrhenius function with acclimation to plant growth temperature (18). The growth temperature was calculated as mean air temperature during the preceding 20 days. Temperature dependence of R_l is calculated using a temperature function used in a model (19, 20). Temperature dependence of K_c, K_o, and Γ_* are modeled based on (21). The parameters used in the temperature functions are deceived in the previous paper (17).

A3-2. Stomatal conductance model

Stomatal conductance (g_{sw}) is calculated based on the semi-empirical model (14).

$$g_{sw} = m_{bb} \frac{A}{c_s} rh_s + b_{bb} \tag{S6}$$

where g_{sw} is stomatal conductance to water vapor, rh_s is relative humidity at the leaf surface, c_s is CO_2 concentration at the leaf surface, m_{bb} is an empirical parameter for a dimensionless slope,

and b_{bb} is an empirical parameter for the zero intercept when net photosynthetic rate is equal or less than zero.

A3-3. Sun/Shade radiation transfer model

Canopy radiation transfer was based on a sun/shade model (15, 21). The direct and diffuse portions of radiation were partitioned based on the method (22), for solving sun/shade radiation transfer model. Photosynthesis and transpiration were separately calculated for sun and shade leaves. An ecosystem-scale parameter of X_{eco} (specifically, Vc_{max25}, J_{max25}, and b_{bb}) can be divided into ecosystem-scale parameters of X_{sun} for sun leaf and X_{shade} for shade leaf.

$$\begin{aligned} X_{eco} &= X_{sun} + X_{shade} \\ &= x_0 L_{sun} + x_{shade} L_{shade} \end{aligned} \quad \text{(S7)}$$

where x_0 are the parameter for unit leaf area, L_{sun} is sunlit leaf area index, and L_{shade} is shaded leaf area index. Assuming the proximity of vertical distribution among leaf nitrogen and irradiance, x_0 is written as:

$$X_{sun} = X_{eco} \frac{\frac{\Omega}{k_n + k_b \Omega L}[1 - \exp(-(k_n + k_b \Omega L))]}{\frac{1}{k_n}[1 - \exp(-k_n)]} \quad \text{(S8)}$$

$$L_{sun} = \frac{[1 - \exp(-k_b \Omega L)]}{k_b} \quad \text{(S9)}$$

$$x_0 = X_{sun} / L_{sun} \quad \text{(S10)}$$

$$x_{shade} = X_{shade} / L_{shade} \quad \text{(S11)}$$

where L is leaf area index, k_b is the extinction coefficient for beam PPFD, k_n is the nitrogen extinction coefficient (21), and Ω is the clumping index (23). The nitrogen extinction coefficient, k_n, was estimated based on a relationship to maximum carboxylation rate per unit leaf area at a top of canopy, vc_{max25} (24). The extinction coefficient, k_b, is calculated as

$$k_b = G(\theta)/\cos\theta \quad \text{(S12)}$$

where G is the G-function defined as the projection coefficient of foliage area on the plane perpendicular to the view direction (25), and θ is the solar zenith angle. We used a look-up-table among G and θ by assuming leaf angle distribution as spherical, planophile, or erectrophile (26).

A3-4. Boundary layer conductance model

Surface meteorological conditions near the big-leaf canopy were estimated from micrometeorological observations. Aerodynamic conductance of sensible heat (g_b; m s-1) was estimated as

$$g_b = 1/(u/u_*^2 + B^{-1}/u_*) \quad \text{(S13)}$$

where u is horizontal wind velocity, u_* is friction velocity, and B^{-1} is the parameter related to the roughness height (dimensionless). According to a method (27), B^{-1} can be estimated using LAI. Once g_b was estimated, leaf temperature was inversely estimated using the bulk surface transfer equation for sensible heat flux. Relative humidity at the leaf surface, rh_s, is calculated based on a method (28).

A3-5. Parameter estimation

The model parameters of Vc_{max25}, ratio of J_{max25} to Vc_{max25} (J_{max25}/Vc_{max25}), m_{bb} and b_{bb} were determined using half-hourly variables: GPP, sensible heat flux, latent heat flux, friction velocity, LAI, atmospheric CO_2 concentration, PPFD, air temperature, relative humidity, wind speed, rainfall, and barometric pressure. The model parameters were determined using a globally optimization method: the shuffled complex evolution method developed at the University of Arizona (SCE-UA; 29, 30, 31). Applying seasonally varying parameters with an 8-day moving window allows consideration of changes in physiological traits, such as periodic drought stress.

Table S1. Responses of CO_2 flux and tree productivities to water conditions. Literatures were surveyed for CO_2 flux in black spruce forests or northern wetlands based on the long-term eddy covariance measurements and radial growth for black spruce trees based on tree ring analyses.

Study site	Vegetation type	Permafrost	Experimental type	Obtained response	References
Interior Alaska, USA	Black spruce forest	permafrost	20-year Eddy covariance	CO_2 sink, GPP, and RE increased with annual total precipitation.	This study
Interior Alaska, USA	Black spruce forest	permafrost	10-year Eddy covariance	CO_2 sink, GPP, and RE increased with annual total precipitation.	33
Central Manitoba, Canada	Black spruce forest	non-permafrost	11-year Eddy covariance	CO_2 sink increased owing to decreased RE in multiple years of high rainfall and resulted in an increase in water table.	34
Central Saskatchewan, Canada	Black spruce forest	non-permafrost	8-year Eddy covariance	CO_2 sink decreased under drought years, because GPP decreased more than RE.	11
Canada, USA	Northern Bog & Fen	non-permafrost	Eddy covaraince at 6 peatlands	Wetter conditions decreased GPP and RE at fens, but increased at bogs.	35
Interior Alaska, USA	Black spurce trees	permafrost &non-permafrost	tree ring at 11 locations	Radial growth positively correlated to annual precipitation.	36
Interior Alaska, USA	Black spurce trees	No information	tree ring at 3 regions	Radial growth positively correlated to precipitation, and negatively correlated to temperature.	37
Interior Alaska, USA	Black spurce trees	permafrost	tree ring at upland and peatland	Radial growth negatively correlated to late summer temperatures in an upland, but were less climate sensitive in a peatland.	38
Eastern Canada	Black spurce trees	No information	Meta analysis of tree ring at 113 sites	Radial growth positively correlated to summer precipitation in previous and current years. Radial growth negatively correlated to summer temperatures at low latitudes, but positively correlated at high latitudes.	39
Central Saskatchewan, Canada	Black spurce trees	No information	tree ring	Precipitation was positively correlated to productivity at a decal scale, and temperature was negatively correlated to productivity. Precipitation was the strong productivity driver.	40
Quebec, Canada	Black spurce trees	No information	tree ring	Cell lumen area positively correlated to precipitation, and/or negatively correlated to daily temperature and VPD.	41

Table S2. Responses of ecosystem productivities to rising atmospheric CO_2 concentration, [CO_2]. Literatures were surveyed for boreal and cool temperate ecosystems or vegetation based on Free Air CO_2 enrichment (FACE), open top chambers, or mesocosm experiments based on the previous studies.

Study site	Vegetation type	Experimental type	Obtained response to enhnaced [CO_2]	References
Finland, Sweden, Netherlands, and Switzerland	Boreal bog	2-year FACE	Vascular plant biomass increased under doubling [CO_2], but the effect was insignificant.	42
Circumboreal Region	Boreal trees	Review	Plant biomass increased with combined increased temperature and elevated [CO_2] despite a decreased maximum carboxylation rate. Water use efficiency increased by elevated [CO_2].	43
Minnesota, USA	Mature trees of black spruce and tamarack	2-year Open top chambers; warming and doubling [CO_2]	Net photosynthesis of black spruce at mean growth temperatures increased with warming under enhanced [CO_2].	44
Minnesota, USA	Understory shrubs (*Chamaedaphne calyculata* and *Rhododendron graenlandicum*)	1-year Open top chambers; warming and doubling [CO_2]	The effects on leaf photosynthesis of enhanced [CO_2] and warming varied by species and seasons. Large enhancements of leaf photosynthesis under enhanced [CO_2] were observed for overwintered leaves across various warming treatments.	45
Minnesota, USA	Understory shrubs, forbs, and graminoid	3-year Open top chambers; warming and doubling [CO_2]	Only minor effects of enhanced [CO_2] on aboveground net primary productivity, LAI, and biodiversity.	46
North West England, UK	Sphagnum mosses	13-week Mesocosm	Elevated [CO_2] increased Sphagnum height and dry weight, and the highest increase was observed under the highest water table. Three-fold increase in the CO_2 sink strength under elevated [CO_2].	47
Northen Japan	Cool temperate forest	11-year FACE	Light compensation point decreased by enhanced [CO_2] for both early successional and late successional trees. Down-regulation was not identified for tree species infected with N-fixing microorganisms.	48

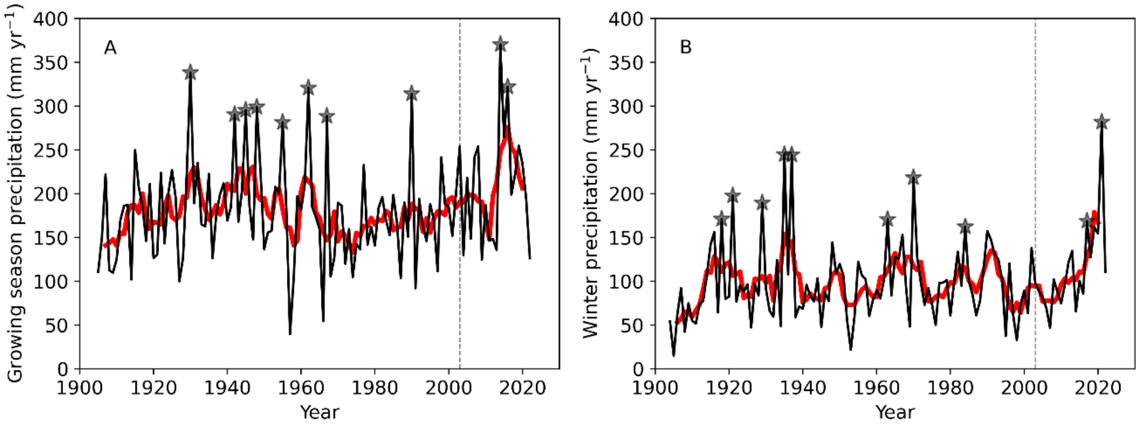

Figure S1. Total precipitation at Fairbanks International Airport according to the National Weather Service for the growing season from May to September (*A*) and for other months (*B*). The black lines represent values for individual years, the red lines represent 5-year moving means, and the stars represent values in the top 10 years from 1900 to 2022. The vertical dashed line represents the year 2003 when the measurement started.

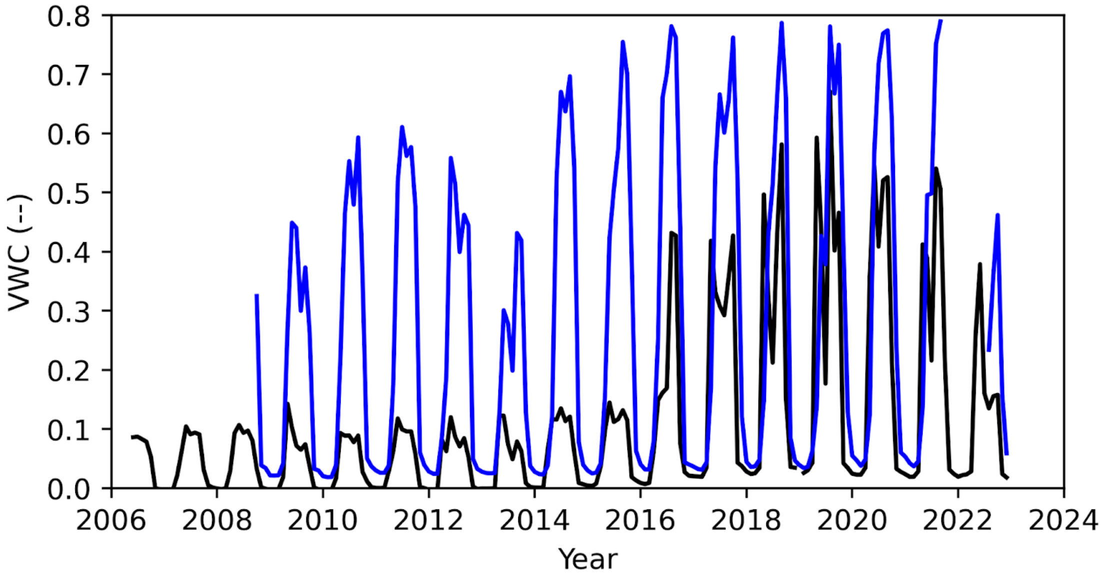

Figure S2. Monthly mean volumetric water content (VWC) at the 0-10 cm depth measured at two locations. The different colors represent the VWC at the different locations. The distance between the two locations is approximately 25 m.

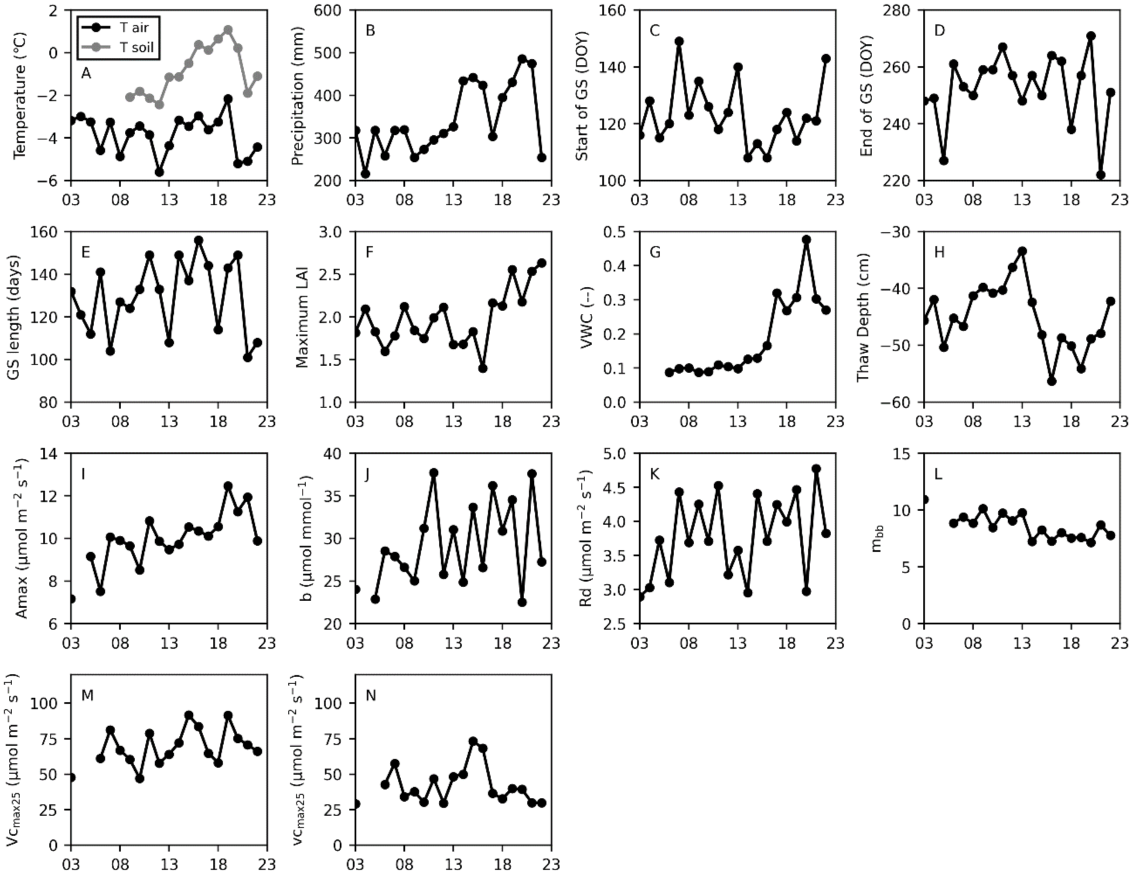

Figure S3. Mean annual air temperature and soil temperature at a depth of 10 cm (*A*), annual precipitation (*B*), start of the growing season (*C*), end of the growing season (*D*), growing season length (*E*), annual maximum LAI (*F*), volumetric water content (VWC) at 0-10 cm from June to July (*G*), annual deepest thaw depth (*H*), annual maximum of monthly mean maximum CO_2 uptake (Amax) (*I*), annual maximum of monthly mean initial slope of CO_2 uptake (b) (*J*), annual maximum of monthly mean dark respiration (Rd) (*K*), the Ball-Berry slope averaged for June and July (*L*), canopy-integrated maximum carboxylation rate at 25 ^{o}C temperature (Vc_{max25}) averaged for June and July (*M*), and leaf-scale maximum carboxylation rate at 25 ^{o}C temperature (vc_{max25}) averaged for June and July (*N*). The growing season was defined as the period when GPP was consecutively higher than 10% of the annual maximum. The photosynthetic parameters (Amax, b, and Rd) were determined daily using a 15-day moving window, based on the relationship between photosynthetically photon flux density and net ecosystem exchange.

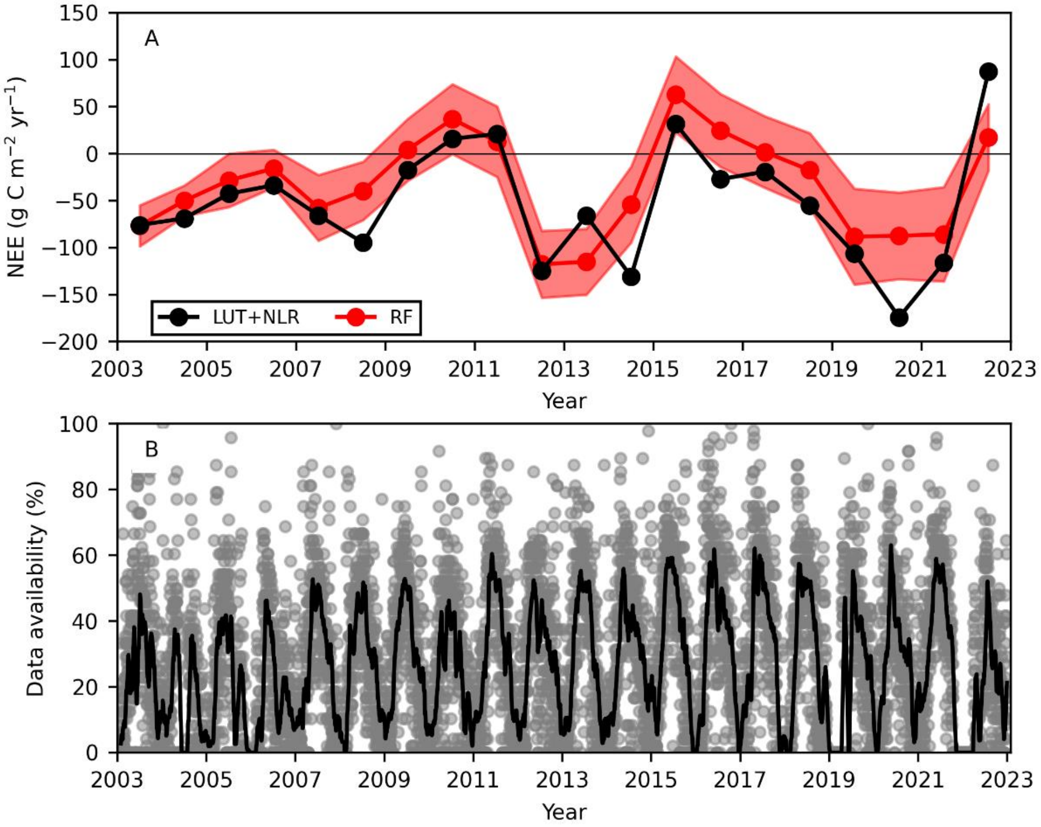

Fig. S4. Interannual variations of NEE gap-filled by two different methods (*A*): 1) combination of look-up table (LUT) and non-linear regression (NLR) and 2) random forest regression (RF). The shadows in (*A*) represent standard error of gap-filled NEE. The standard error for NEE by LUT and NLR was calculated based on 100 bootstrapped samples, which was too small to be resolved in the figure. The standard error for NEE for the random forest regression was calculated using 20 different regressions that had different hyper-parameters determined from the 20 different initial parameters. The data availability for NEE (*B*), where dots represent the daily value, and the line represents the 30-day moving mean.

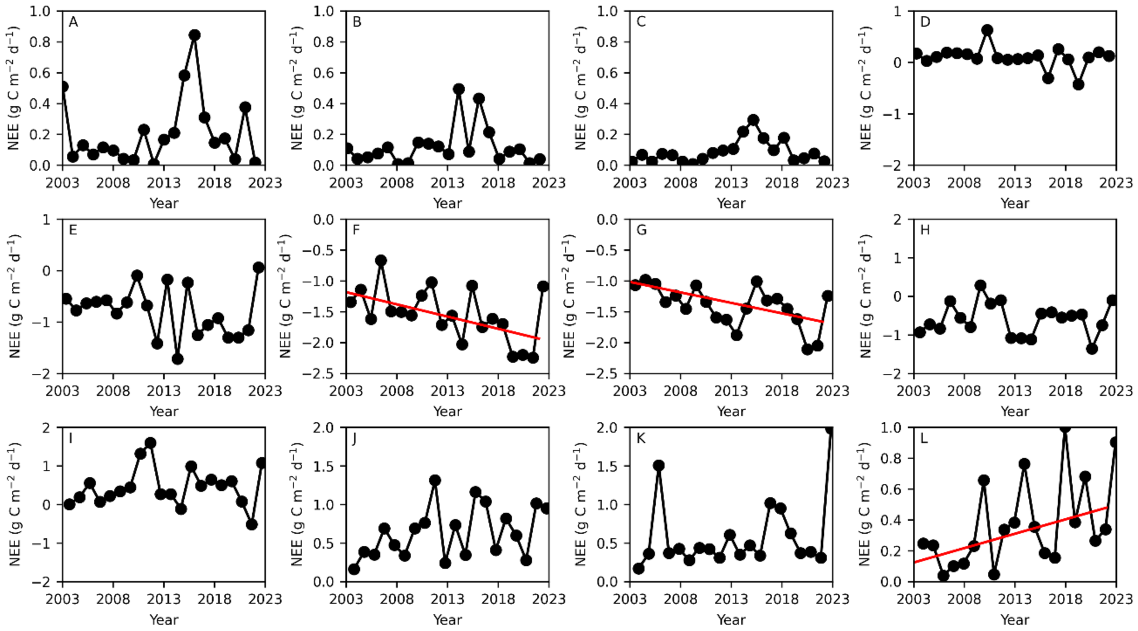

Figure S5. Monthly mean NEE in January (*A*), February (*B*), March (*C*), April (*D*), May (*E*), June (*F*), July (*G*), August (*H*), September (*I*), October (*J*), November (*K*), and December (*L*). The dashed line in (b) represents the linear regression, where the p value was calculated based on the Mann–Kendall test, and the shaded area represents the 95% prediction interval. The trend line based on the Theil-Sen slope is only shown when the p values were greater than 0.05.

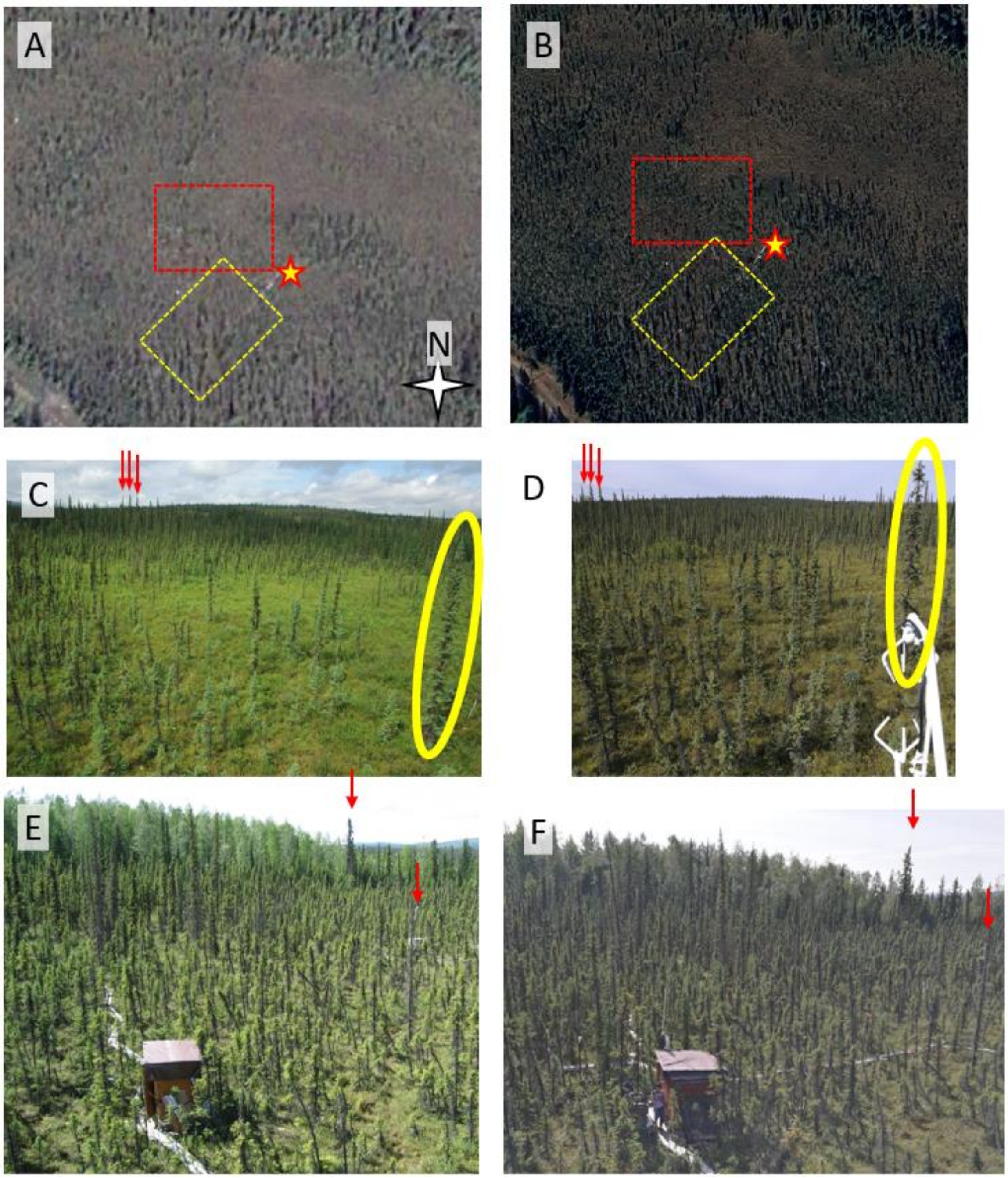

Figure S6. Aerial photograph around the tower site based on Google Earth (*A*, *B*) and photos taken from the tower (*C*, *D*, *E*, *F*) for comparing the vegetation change from 2003 to present. The panels *A*, *C* and e show the conditions in the early study period: the aerial photograph by Google Earth was taken on June 19, 2003 (*A*) and the photos were taken on July 10, 2005 (*C*), and June 8, 2007 (*E*). The panels *B*, *D*, and f show the conditions in the last study period: the aerial photograph by Google Earth was taken on May 27, 2023 (*B*), and the photos were taken on August 12, 2019 (*D*, *F*). Red dashed squares in (*A*, *B*) are areas for (*C*, *D*), and yellow dashed squares in (*A*, *B*) are the area taken for (*E*, *F*). Yellow circles and red arrows represent landmarks for comparing the paired photos. Note that the dominant wind direction was northwest.

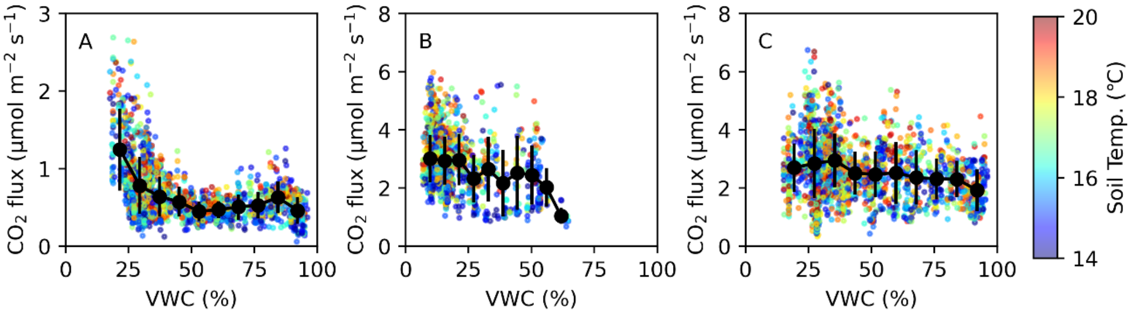

Figure S7. Relationships between volumetric water content (VWC) at the 0-10 cm depth measured at for *Sphagnum* mosses (*A*), and *Carex* spp. (*B*, *C*) based on automated closed chambers from 2016 to 2018. The data are shown for soil temperature ranging from 14 °C to 20 °C at the 10 cm depth. The soil respiration was measured using a dark chamber. Individual data points represent measurements taken at half-hourly interval.

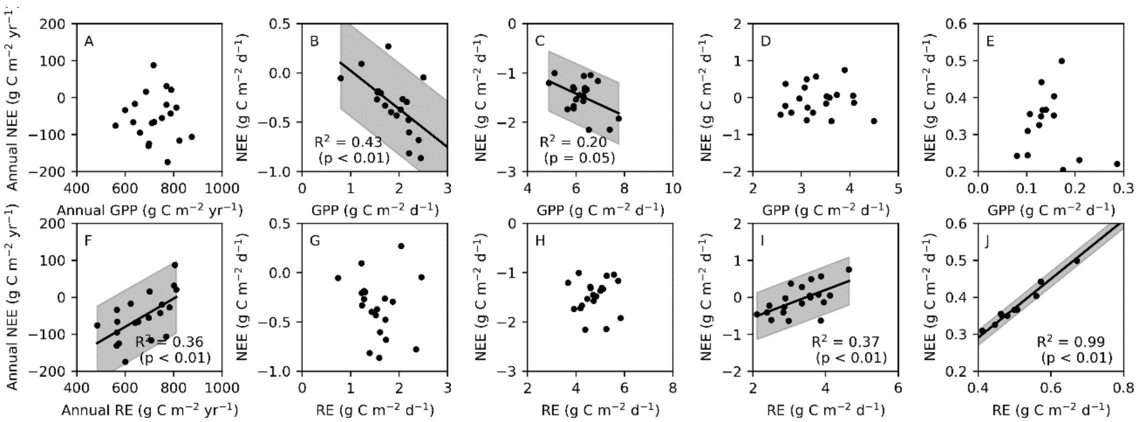

Figure S8. Relationships between NEE and GPP (*A*, *B*, *C*, *D*, *E*) and between NEE and RE (*F*, *G*, *H* ,*L*, *J*) for the annual mean (*A*, *F*), April-May (*B*, *G*), June-July (*C*, *H*), August-September (*D*, *J*), and October-March (*E*, *J*).

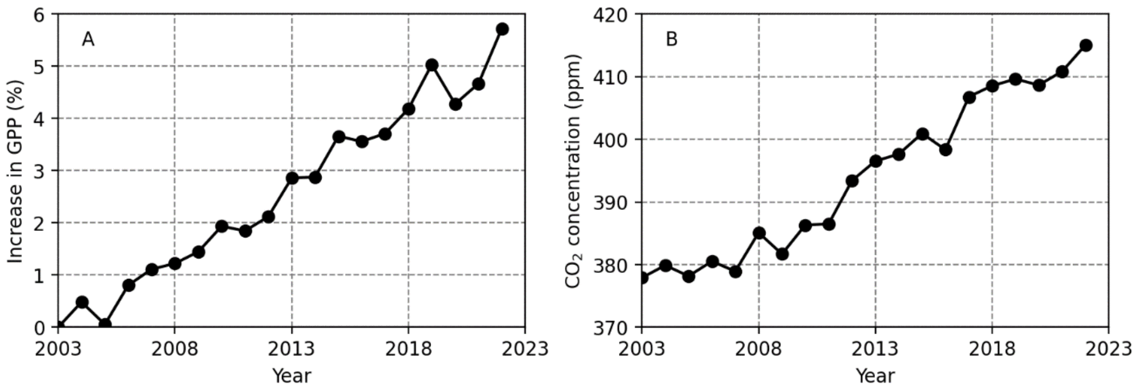

Figure S9. Estimated fractional increase in annual gross primary productivity (GPP) associated with rising atmospheric CO_2 concentration (*A*) and atmospheric CO_2 concentration in June based on aircraft measurements at Poker Flat near Fairbanks, operated by the National Oceanic and Atmospheric Administration (32) (*B*). The increase in GPP resulting from the rising CO_2 concentrations was estimated based on iBLM-EC version 2 (8).

SI References

1. M. Ueyama et al., Influences of various calculation options on heat, water, and carbon fluxes determined by open- and closed-path eddy covariance method. Tellus B **64**, 19048 (2012).
2. M. Ueyama, H. Iwata, Y. Harazono, Autumn warming reduces the CO_2 sink of a black spruce forest in interior Alaska based on a nine-year eddy covariance measurement. Glob. Change Biol. **20**, 1161-1173 (2014).
3. E. K. Webb, G. L. Pearman, R. Leuning, Correction of flux measurements for density effects due to heat and water vapour transfer. Quart. J. Roy Meteorol. Soc. **106**, 85-100 (1980).
4. W. J. Massman, A simple method for estimating frequency response corrections for eddy covariance system. Agric. For. Meteorol. **104**, 182-198 (2000).
5. P. Hignett, Corrections to temperature measurements with a sonic anemometer. Bound. Lay. Meteorol. **61**, 175-187 (1992).
6. G. Jocher, et al., Apparent winter CO_2 uptake by a boreal forest due to decoupling. Agric. For. Meteorol. **232**, 23-34 (2017).
7. E. Falge et al., Gap filling strategies for defensible annual sums of net ecosystem exchange. Agric. For. Meteorol. **107**, 43-69 (2001).
8. M. Ueyama et al., Inferring CO_2 fertilization effect based on global monitoring land-atmosphere exchange with a theoretical model. Environ. Res. Lett. **15**, 084009 (2020).
9. G. G. Burba, D. K. McDermitt, A. Grelle, D. J. Anderson, L. Xu, Addressing the influence of instrument surface heat exchange on the measurements of CO_2 flux from open-path gas analyzers. Glob. Change Biol. **14**, 1854-1876 (2008).
10. B. Amiro, Estimating annual carbon dioxide eddy fluxes using open-path analyser for cold forest sites. Agric. For. Meteorol. **150**, 1366-1372 (2010).
11. P. Krishnan, T. A. Black, A. G. Barr, N. J. Grant, D. Gaumont-Guay, Z. Nesic, Factors controlling the interannual variability in the carbon balance of a southern boreal black spruce forest. J. Geophys. Res. Atm. **113**, D09109 (2008).
12. P. Jarvis, S. Linder, Constraints to growth of boreal forests. Nature **405**, 904-905 (2000).
13. G. D. Farquhar, S. von Caemmerer, J. A. Berry, A biochemical model of photosynthesis CO_2 assimilation in leavers of C3 species. Planta **149**, 78-90 (1980).

14. J. T. Ball, I. E. Woodrow, J. A. Berry, A model predicting stomatal conductance and its contribution to the control of photosynthesis under different environmental conditions progress Photosynth. Res. ed I Biggins (Dordrecht: Martinus-Nijhoff), pp. 221-224 (1987).
15. Y. Ryu et al., Integration of MODIS land and atmosphere products with a coupled-process model to estimate gross primary productivity and evapotranspiration from 1 km to global scales. Glob. Biogeochem. Cycles **25**, doi:10.1029/2011GB004053 (2011).
16. M. Ueyama, N. Tahara, H. Nagano, N. Makita, H. Iwata, Y. Harazono, Leaf- and ecosystem-scale photosynthetic parameters for the overstory and understory of boreal forests in interior Alaska. J. Agric. Meteorol. **74**, 79-86 (2018).
17. M. Ueyama et al., Optimization of biochemical model with eddy covariance measurements in black spruce forests of Alaska for estimating CO_2 fertilization effects. Agric For. Meteorol. **222**, 98-111 (2016).
18. J. Kattge, W. Knorr, Temperature acclimation in a biochemical model of photosynthesis: a reanalysis of data from 36 species. Plant, Cell Environ. **30**, 1176-1190 (2007).
19. C. J. Bernacchi, C. Pimentel, S. P. Long, *In vivo* temperature response functions of parameters required to model RuBP-limited photosynthesis. Plant, Cell Environ. **26**, 1419-1430 (2003).
20. C. J. Bernacchi, E. L. Singsaas, C. Pimentel, A. R. Portis Jr, S. P. Long, Improved temperature response functions for models of Rubisco-limited photosynthesis. Plant, Cell Environ. **24**, 253-259 (2001).
21. D. G. G. de Pury, G. D. Farquhar, Simple scaling of photosynthesis from leaves to canopies without the errors of big-leaf models. Plant, Cell Environ. **20**, 537-557 (1997).
22. A. Weiss, J. M. Norman, Partitioning solar radiation into direct and diffuse, visible and near-infrared components. Agric. For. Meteorol. **34**, 205-213 (1985).
23. L. He, J. M. Chen, J. Pisek, C. B. Schaaf, A. H. Strahler, Global clumping index map derived from the MODIS BRDF product. Remote Sens. Environ. **119** 118-130 (2012).
24. J. Lloyd et al., Optimization of photosynthetic carbon gain and within-canopy gradients of associated foliar traits for Amazon forest trees. Biogeosciences **7**, 1833-1859 (2010).
25. J. Ross, The radiation regime and architecture of plant stands. Junk Publishers, The Hague, 391 pp (1981).
26. J. Pisek et al., Intercomparison of clumping index estimates from POLDER, MODIS, and MISR satellite data over reference sites. ISPRS J. Photo. Remote Sens. **101** 47-56 (2015).
27. J. P. Lhomme, A. Chehbouni, B. Monteny, Sensible heat flux-radiometric surface temperature relationship over sparse vegetation: parameterizing B^{-1}. Boundary-Layer Meteorol. **97**, 431-457 (2000).
28. H. B. Su, K. T. Paw U, R. H. Shaw, Development of a coupled leaf and canopy model for the simulation of plant-atmosphere interaction. J. Appl. Meteorol. **35**, 733-748 (1996).
29. Q. Y. Duan, V. K. Gupta, S. Sorooshian, Shuffled complex evolution approach for effective and efficient global minimization. J. Optim. Theor. Appl. **76**, 501-521 (1993).
30. Q. Y. Duan, S. Sorooshian, V. K. Gupta, Effective and efficient global optimization for conceptual rainfall-runoff models. Water Resour. Res. **28**, 1015-1031 (1992).
31. Q. Y. Duan, S. Sorooshian, V. K. Gupta, Optimal use of the SCE-UA global optimization method for calibrating watershed models. J. Hydrol. **158**, 265-284 (1994).
32. K. McKain et al., Global greenhouse gas reference network flask-air PFP sample measurements of CO_2, CH_4, CO, N_2O, H_2, SF_6 and isotopic ratios collected from aircraft vertical profiles [Data set]. Version: 2023-08-11 (2023).
33. E. Euskirchen et al., Persistent net release of carbon dioxide and methane from an Alaskan lowland boreal peatland complex. Glob. Change Biol. **30**, e17139 (2024).
34. A. L. Dunn, C. C. Barford, S. C. Wofsy, M. L. Goulden, B. C. Daube, A long-term record of carbon exchange in a boreal black spruce forest: means, responses to interannual variability, and decadal trends. Glob. Change Biol. **12**, 1-14 (2006).
35. B. N. Sulman et al., CO_2 fluxes at northern fens and bogs have opposite responses to inter-annual fluctuations in water table. Geophys. Res.Lett. **37**, L19702 (2010).

36. M. Ueyama et al., Does summer warming reduce black spruce productivity in interior Alaska? J. For. Res. **20**, 52-59 (2015).
37. X. J. Walker, M. C. Mack, J. F. Johnstone et al., Stable carbon isotope analysis reveals widespread drought stress in boreal black spruce forests. Glob. Change Biol. **21**, 3102-3113 (2015).
38. M. Wilmking, I. Myers-Smith, Changing climate sensitivity of black spruce (*Picea mariana* Mill.) in a peatland–forest landscape in Interior Alaska. Dendrochronologia **25**, 167-175 (2008).
39. C. Chagnon, A. R. Wotherspoon, A. Achim, Deciphering the black spruce response to climate variation across eastern Canada using a meta-analysis approach. For. Ecol. Manage. **520**, 120375 (2022).
40. O. Bouriaud, D. Frank, J. S. Bhatti, Assessing the influence of climate-water table interactions on jack pine and black spruce productivity in western central Canada. Ecoscience **21**, 315-326 (2014)
41. P. F. Puchi, D. Castagneri, S. Rossi, M. Carrer, Wood anatomical traits in black spruce reveal latent waterconstraints on the boreal forest. Glob. Change Biol. **26**, 1767-1777 (2020).
42. M. R. Hoosbeek, N. van Breemen, F. Berendse, P. Grosvernier, H. Vasander, B. Wallén, Limited effect of increased atmospheric CO_2 concentration on ombrotrophic bog vegetation. New Phytol. **150**, 459-463 (2001).
43. J. R. Stinziano, D. A. Way, Combined effects of rising [CO_2] and temperature on boreal forests: growth, physiology and limitations. Botany **92**, 425-436 (2014).
44. M. E. Dusenge et al., Boreal conifers maintain carbon uptake with warming despite failure to track optimal temperatures. Nat. Com. **14**, 4667 (2023).
45. E. J. Ward et al., Photosynthetic and respiratory responses of two bog shrub species to whole ecosystem warming and elevated CO_2 at the boreal-temperate ecotone. Front. For. Glob. Change **2**, 54 (2019).
46. M. Y. McPartland, R. A. Montgomery, P. J. Hanson, J. R. Phillips, R. Kolka, B. Palik, Vascular plant species response to warming and elevated carbon dioxide in a boreal peatland. Environ. Res. Lett. **15**, 124066 (2020).
47. T. R. Newman, N. Wright, B. Wright, S. Sjögersten, Interacting effects of elevated atmospheric CO_2 and hydrology on the growth and carbon sequestration of Sphagnum moss. Wetlands Eco. Manage. **26**, 763-774 (2018).
48. T. Koike et al., Ecophysiology of deciduous trees native to Northeast Asia grown under FACE (Free Air CO_2 Enrichment), J. Agric. Meteorol. **71**, 174-184 (2015).
